# The cost of healthy eating in two major cities in Ecuador: a comparative analysis

**DOI:** 10.3389/fnut.2025.1516106

**Published:** 2025-09-26

**Authors:** Leidy Gonzabay-Parrales, Lesly Alay Chimborazo, Giuliana Altamirano Flores, Diana Ocaña Grijalva, Doménica Arias Cárdenas, Jair Gómez Rubiano, Valeria Hernández Andrade, Nathalia Pérez Molina, Martín Terán Navas, Camila Contero Gómez, Fatima Romo Guaranda, Cecilia Alejandra García Ríos, Jose E. Leon-Rojas

**Affiliations:** ^1^NeurALL Research Group, Quito, Ecuador; ^2^Escuela de Medicina, Universidad Católica de Santiago de Guayaquil, Guayaquil, Ecuador; ^3^Escuela de Nutrición, Escuela Superior Politécnica del Litoral, Guayaquil, Ecuador; ^4^Carrera de Medicina, Facultad Ciencias de la Salud, Universidad Nacional de Chimborazo, Riobamba, Ecuador; ^5^Facultad de Medicina, Universidad de las Américas (UDLA), Quito, Ecuador

**Keywords:** healthy diet, regular diet, cost, affordability, Ecuador

## Abstract

**Background/objectives:**

Healthy eating is essential to maintaining health and preventing disease. However, various economic and social factors make it difficult to access an adequate diet in many regions, especially in low-middle income countries (LMIC). In Ecuador, the economy underwent significant changes following the SARS-COV-2 pandemic, affecting food prices and, therefore, the population’s ability to maintain a healthy diet. We want to showcase the costs of a healthy diet in Quito and Guayaquil by evaluating the price of food items sold to consumers in major supermarket chains/food suppliers.

**Methods:**

A diet model was designed based on foods from the basic family basket (BFB) and standard nutritional recommendations. Prices were collected through visits to supermarkets and 3 types of diet were analysed: regular diet with BFB portions, regular diet with healthy portions, and our healthy diet model.

**Results:**

The cost of a healthy diet is significantly higher than a regular diet; with the price of healthy eating in Ecuador, in 2023, being $184.66 per person per month, which represents 41% of the unified basic salary (or 3.2 times more expensive than the BFB), making it unaffordable for many families with scarce resources. In Quito and Guayaquil, the most expensive foods in a healthy diet were dairy products, eggs, and meat.

**Conclusion:**

Healthy eating in the two major cities of Ecuador represents almost half of the basic monthly salary, making it inaccessible to most families with limited resources, and becoming a matter of public health. Our study highlights the need for public policies to improve access to healthy foods as well as local policies to incentivize direct trade of food items (i.e., directly from the producer to the final consumer).

## 1 Introduction

Healthy eating emerges as a fundamental pillar, from a medical perspective, to preserve health by providing substantial nutritional support and playing a crucial role in preventing multiple diseases throughout life. In the systematic analysis of dietary risk conducted by Afshin et al. (36) across 195 countries from 1990 to 2017, poor diets were estimated to be responsible for approximately 11 million deaths, primarily due to cardiovascular disease and cancer (36). The leading dietary risk factors included high sodium intake, low consumption of whole grains and insufficient fruit intake (36). Furthermore, in a large cohort study published by Shan et al. (1), greater adherence to a healthy eating pattern was significantly associated with a reduction in the risk of coronary heart disease and stroke by 10–20% over up to 32 years of follow-up [HR 0.80 (95% CI, 0.77–0.830)] (1). On the other hand, in the systematic review published in 2019 by Lassale et al. (2) it was found that an inversely proportional relationship exists between a healthy diet (especially the traditional Mediterranean diet) and depression. Furthermore, in the meta-analysis presented in 2017 by Kelly et al. with 15,285 patients from 7 studies, a statistically significant association was found between a healthy diet and decreased mortality in patients with chronic kidney disease (3).

Despite the clear and widely recognized importance of healthy eating, various obstacles, such as rapid urbanization, lifestyle changes, economic instability, and the recent SARS-COV-2 pandemic, have affected the population’s ability to maintain an adequate diet; in particular, low-middle income countries (LMIC) (4, 5). In Ecuador, the average per capita caloric intake is 2,141 kilocalories per day, which corresponds to the minimum estimated threshold required to accomplish basic energy requirements. Nevertheless, this average masks significant disparities in both food distribution and nutritional quality, which are reflected in alarming public health indicators; for instance, the prevalence of chronic malnutrition among children stands at 25.3% (6). Additionally, according to the 2015 report by the Food and Agriculture Organization (FAO), 11% of Ecuadorians lacks adequate access to food. This situation is closely linked to structural factors such as poverty, which affects 25.8% of the population, and limited the accessibility to a healthy diet. These conditions contribute to ongoing food and nutrition insecurity in Ecuador (7).

Moreover, data from the National Health and Nutrition Survey (ENSANUT-ECU) in 2012 reveal critical deficiencies in dietary patterns. On average, Ecuadorians consume only half of the daily recommended intake of fruits. Similarly, protein consumption is low and does not meet daily needs. In contrast, the intake of carbohydrates and fats exceeds recommended levels. These nutritional gaps are more pronounced among households in the lowest quintile, underscoring the strong correlation between socioeconomic status and the fulfilment of daily dietary requirements (8).

On the other hand, the World Health Organization (WHO) notes that after years of global “stability,” the world’s percentage of people experiencing hunger increased dramatically in 2020 and continued to rise in 2021, reaching 9.8%; this reflects the negative impact of the pandemic on the global economy and people’s quality of life (5). In the Latin American context, the Pan American Health Organization (PAHO) highlights that 22.5% of the population in the region does not have sufficient resources to access a healthy diet, with economic and social factors being the main determinants (4).

As for Ecuador, after the pandemic the economy experienced substantial transformations, including an increase in the Consumer Price Index (CPI) and a monthly inflation of 0.36%, directly affecting the costs of basic food items (9, 10); furthermore, Ecuador’s annual inflation rate has experienced significant fluctuations: in 2021 this rate was 0.13%, followed by 3.47% during 2022 and 2.22% in 2023 (11). This economic impact might have limited the access to essential foods for a balanced diet for the population, generating the need for a comparative analysis between the costs of a healthy diet and the costs of a regular diet according to the basic family basket (BFB) established by the Ecuadorian government. Therefore, in this context, our research focuses on determining the real costs of a healthy diet in Ecuador, specifically in Quito and Guayaquil, to determine the accessibility for a family of two adults.

## 2 Materials and methods

We conducted a descriptive cross-sectional study by applying a healthy diet model that was designed based on foods reported from the Ecuadorian basic family basket (BFB) and by considering standard nutritional recommendations. The BFB was created by the Ecuadorian government and the National Institute of Statistics and Census (INEC) and is defined as a set of goods and services that are essential to meet the basic needs of a household composed of 4 members with 1.6 income earners, who earn the unified basic salary ($450 USD as of 2023). The foods and their respective categories included in the BFB are presented in Table 1 (12).

**Table 1:** Comparison of basic family basket (BFB) and healthy diet models adjusted to fulfil 90–105% of the daily requirements: food items and portions for two people, for one month.

Category	BFB model (Characteristics of collected information)	Healthy model (Characteristics of collected information)	Monthly amount based in BFB portions for two people	Monthly amount of healthy portions for two people
Grains and derivatives	White rice	Brown rice	500 grams	2,240 grams
	White wheat tagliatelle (egg-free)	Whole wheat tagliatelle noodles	500 grams	1493.36 grams
	White loaf bread with crust	Whole wheat loaf bread with crust	500 grams	1493.36 grams
Meat	Meat with bone		500 grams	
	Meat without bone, brisket or steak cut	Beef loin cut	500 grams	1920 grams
	Chicken (different of skinless)	Skinless chicken breast	500 grams	3,840 grams
Fish and seafood	Fish (Albacore, tilapia, dorado and snook)	Corvina fish	500 grams	1,600 grams
	Canned tuna with oil	Canned tuna in water (sodium <10% of the daily value)	92 grams	1,600 grams
Edible fats and oils	Vegetable palm oil	Cold-pressed olive oil (dark glass bottle)	500 milliliters	480 grams
	Vegetable shortening (margarine)		250 grams	
Milk, dairy products and eggs	Chicken egg	Chicken egg	500 grams	3,080 grams
	Whole milk	Semi-skimmed milk	500 milliliters	21,000 millilitres
	Fresh cheese	Fresh cheese	500 grams	4,400 grams
Fruits	Avocado	Avocado	500 grams	1493.36 grams
	Lemon	Lemon	500 grams	1493.36 grams
	Orange	Orange	500 grams	1493.36 grams
	Banana	Banana	500 grams	1493.36 grams
	Naranjilla	Naranjilla	500 grams	1493.36 grams
	Plantain	Plantain	500 grams	1920 grams
Vegetables	Green peas	Fresh green peas	500 grams	1920 grams
	White onion	White onion	500 grams	1920 grams
	Red onion	Red onion	500 grams	1920 grams
	Corn	Corn	500 grams	1920 grams
	Beans	Fresh beans	500 grams	1920 grams
	Fava beans	Fresh fava beans	500 grams	1920 grams
	Tomatoes	Tomatoes	500 grams	1920 grams
Tubers and derivatives	Potatoes	Potatoes	500 grams	12,600 grams
	Yucca	Yucca	500 grams	12,600 grams
Legumes and derivatives	Lentils	Lentil	500 grams	8,960 grams
	Dry beans	Dry beans	500 grams	4,480 grams
	Peanuts		500 grams	
Coffee, tea and soft drinks	Sugar	White sugar	500 grams	2,400 grams
	Salt	Common salt	1,000 grams	280 grams
	Instant coffee	Artisanally ground coffee beans	500 grams	990 grams
	Gelatin powder	Unflavored gelatin powder (High in protein, low in sugar, and free of trans fats)	42.5	40 gr

The representation of a healthy plate, according to food-based dietary guidelines of Ecuador (GABA, according to its acronym in English), is depicted in the shape of a wooden spoon, symbolizing the integration of essential foods for a balanced and culturally appropriate diet that promotes healthy eating habits. This spoon visually illustrates the 11 recommendations for a healthy diet and lifestyle (6). The spoon bowl is divided into four sections: the green section (approximately 50%) corresponds to fruits and vegetables such as bananas and tomatoes; the blue section (approximately 20%) emphasizes grains and cereals like rice and potatoes; the purple section (approximately 20%) represents protein sources like eggs and chicken; and the beige section (approximately 10%) depicts fat-rich foods like avocado (6). Additionally, the handle of the spoon emphasizes commensality, promoting Ecuadorian food production, as well as the importance of safe drinking water. The outer edge of the spoon promotes physical activity, including running and swimming. Besides, a separate circular area highlights foods that should be avoided, such as candies. Some of these characteristics of the spoon, along with the 11 recommendations, were considered in the development of our diet (6). In this regard, we created our healthy diet model based on the following standard nutritional recommendations: 2000 kcal per day divided into 55% carbohydrates, 20% protein and 25% fat (13, 14). The portion sizes in the diet were established from the WHO, other international agencies and food-based dietary guidelines of Ecuador (GABA) whose portions are shown in Table 2 (15–18).

**Table 2:** Portion guidelines and serving frequency for a healthy diet.

Food item	Recommended portion	Number of servings
Cereals	40 grams	2 servings/day
Chicken meat	120 grams	4 servings/week
Beef	120 grams	2 servings/week
Fish	100 grams	2 servings/week
Canned tuna	100 grams	2 servings/week
Fats and oils	5 grams	2 servings/day
Milk	250 milliliters	3 servings/day
Cheese	44 grams	2 servings/day
Eggs	55 grams	1 serving/day
Fruits	80 grams	2 servings/day
Vegetables	80 grams	3 servings/day
Tubers	150 grams	2 servings/day
Legumes	80 grams	3 servings/day
Sugar	15 grams/day	Not applicable
Salt	5 grams/ day	Not applicable
Coffee	80 grams	4 servings/day
Gelatine	240 mL	1 serving/week

With this data, a diet adjustment was made to fulfil 90 to 105% of the daily nutritional requirements for a complete month for two average adults consuming 2000 kcal per day each, considering that 1 gram of carbohydrate is equivalent to 4 kcal, 1 gram of protein is equivalent to 4 kcal and 1 gram of fat is equivalent to 9 kcal. As part of the adjustments, we used raw versions of protein items and cooked versions of carbohydrate foods. For the diet adjustment, we based on “Ecuadorian Food Exchange List” of the dietary guidelines of Ecuador (6). The portions are shown in Table 1, along with the healthy version of each food chosen in our model.

Evidently, not all the products listed in the BFB could be considered as part of a healthy diet; therefore, we removed margarine and soda drinks, similarly peanuts were removed because they exceeded the recommended daily fat percentage, and other products were exchanged for their healthier counterparts. Additionally, we also modified the BFB diet into a “healthy BFB diet,” taking into consideration the average Ecuadorian diet and portions considered in the BFB but including healthy options of each food item based in evidence (Table 2) (19–26). Therefore, for our analysis, we created three types of diet: a regular diet according to BFB recommended portions, regular diet with healthy portions (healthy BFB), and our healthy diet model for two adults for one month; we also report the costs of alimentation according to INEC (open data published) adjusted for two people (https://www.ecuadorencifras.gob.ec/informacion-historica-ipc-canastas-2023/).

The lowest and highest prices of the regular and healthy versions of the aforementioned food items were collected during field visits to six supermarkets in both Guayaquil and Quito (the two most densely populated cities of Ecuador); each supermarket was identified by a letter in order to preserve anonymity. The sample included a mix of high-end, mid-range and low-end supermarket chains to obtain diverse data about prices in urban areas. Supermarkets B and D were classified as lower mid-range, supermarket F as low-end, C as mid-range, E as upper mid-range and supermarket A as high-end. It is important to consider that the classification of supermarkets by market tier refers to their general pricing strategy, target consumer base, and product variety.

To identify the cheapest and most expensive options of every food item, each data collector divided the product’s price by its weight in grams to calculate the cost per gram. This approach ensured that differences in package sizes did not interfere with accurately determining the lowest and highest price of each product. We determined specific characteristics of the products to avoid significant price, as detailed in Table 1. The collector recorded both the minimum and maximum costs, along with their corresponding weights in grams. This process was carried out for every food item in each supermarket. The data was collected using the Kobo ToolBox software, after which the information was downloaded into an Excel spreadsheet for data handling and quality checks. For comparison with official data, we used the value of alimentation expenditure component of the BFB to September 2023 obtained from the website of the INEC.

We calculated the average cost of the healthy diet overall and for each city. We determined the monthly and daily cost of a healthy diet for one person. To assess affordability, we considered the premise that, according to previous studies, an affordable diet must represent an expense of less than 30% of the family’s income (27). In this context, we calculated the ratios between the cost of the diets (healthy and regular) and the basic monthly salary of a person for September, 2023 in Ecuador. Additionally, we compared the monthly cost of the healthy diet for one person with income quintiles based on the latest published information.

## 3 Results

Our analysis of the data collected reveals an important gap between the affordability of a regular diet and a healthy diet in both Quito and Guayaquil.

**Figure 1:**
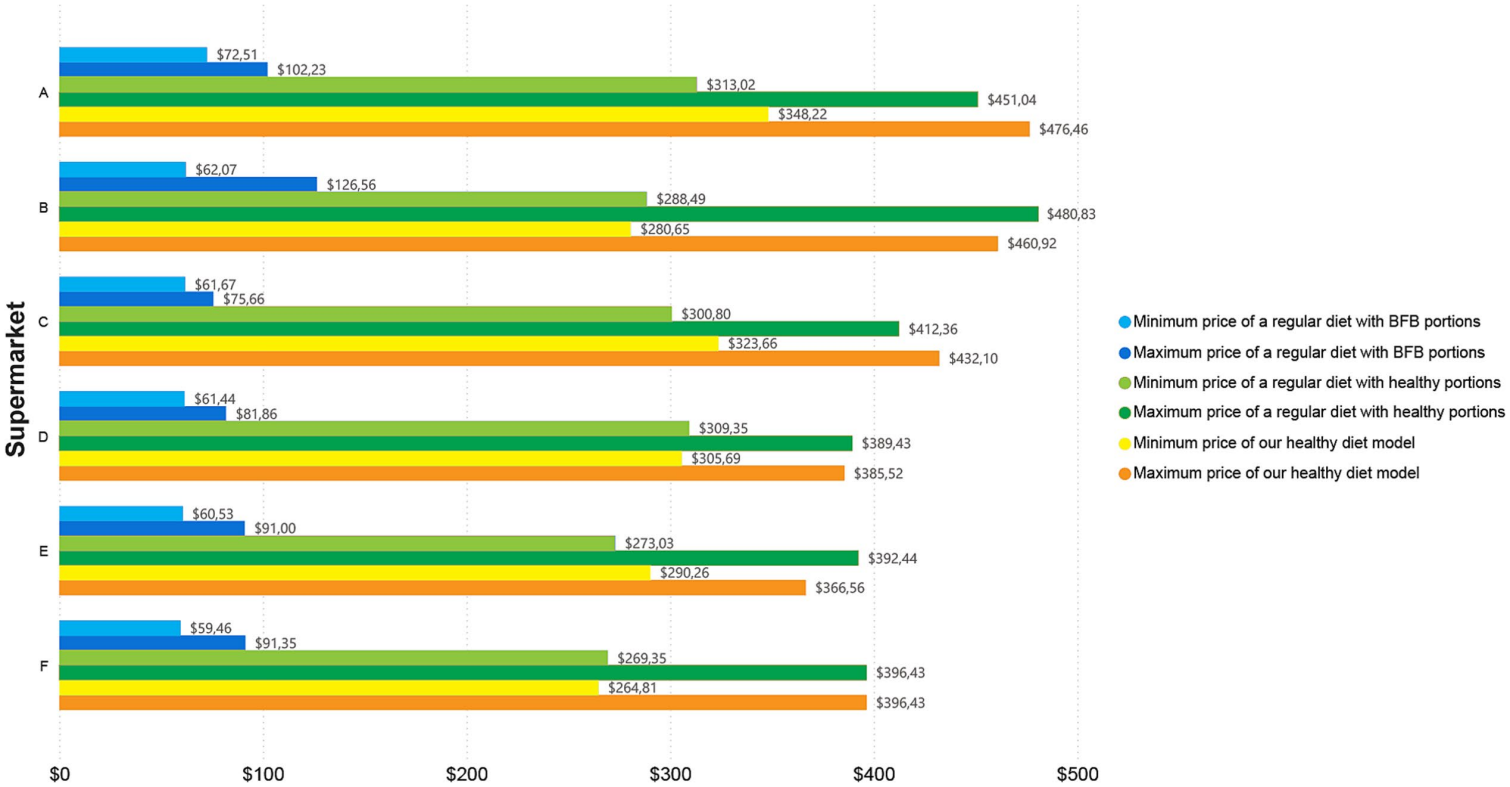
Overall price by supermarket, showcasing minimum and maximum cost of the three analysed diets per month for two adults.

Regarding the analysis of diets by supermarket, it is noted that, in all supermarkets, our healthy diet model was more expensive compared to the other diets. In addition, the cost of the regular diet with BFB portions is much lower than the other diets; when looking at the minimum cost analysis for this diet, supermarket F (low-end) offered the most economical option with $59.46, while supermarket A (high-end) was the most expensive with $72.51. In comparison, when looking at the maximum cost, supermarket C (mid-range) was the most economical and supermarket B (lower mid-range) was the most expensive with $75.66 and $126.56, respectively. On the other hand, for our healthy diet model, when looking into the minimum cost analysis, the most expensive option was supermarket A (high-end) with $348.22 and the most economical was F (low-end) with $264.81; while, in terms of maximum cost, the most expensive supermarket was A (high-end) with $476.46 and the most economical was supermarket E (upper mid-range) $366.56. The minimum and maximum costs for the three analysed diets per month for two adults, stratified by supermarket can be found in Figure 1.

**Figure 2:**
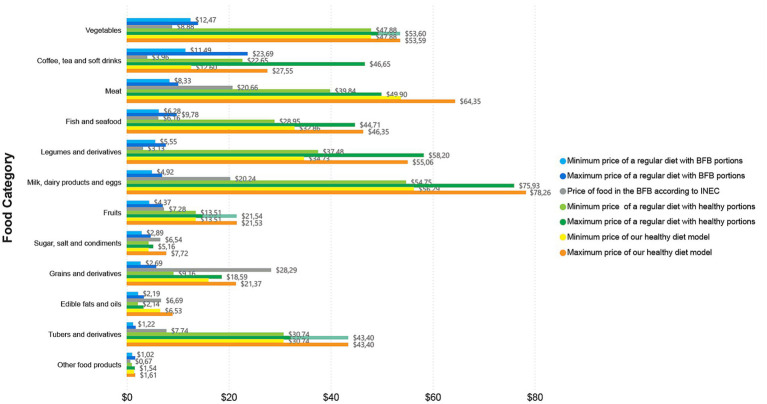
Overall price by food category, showcasing minimum and maximum cost of the three analysed diets per month for two adults.

**Figure 3:**
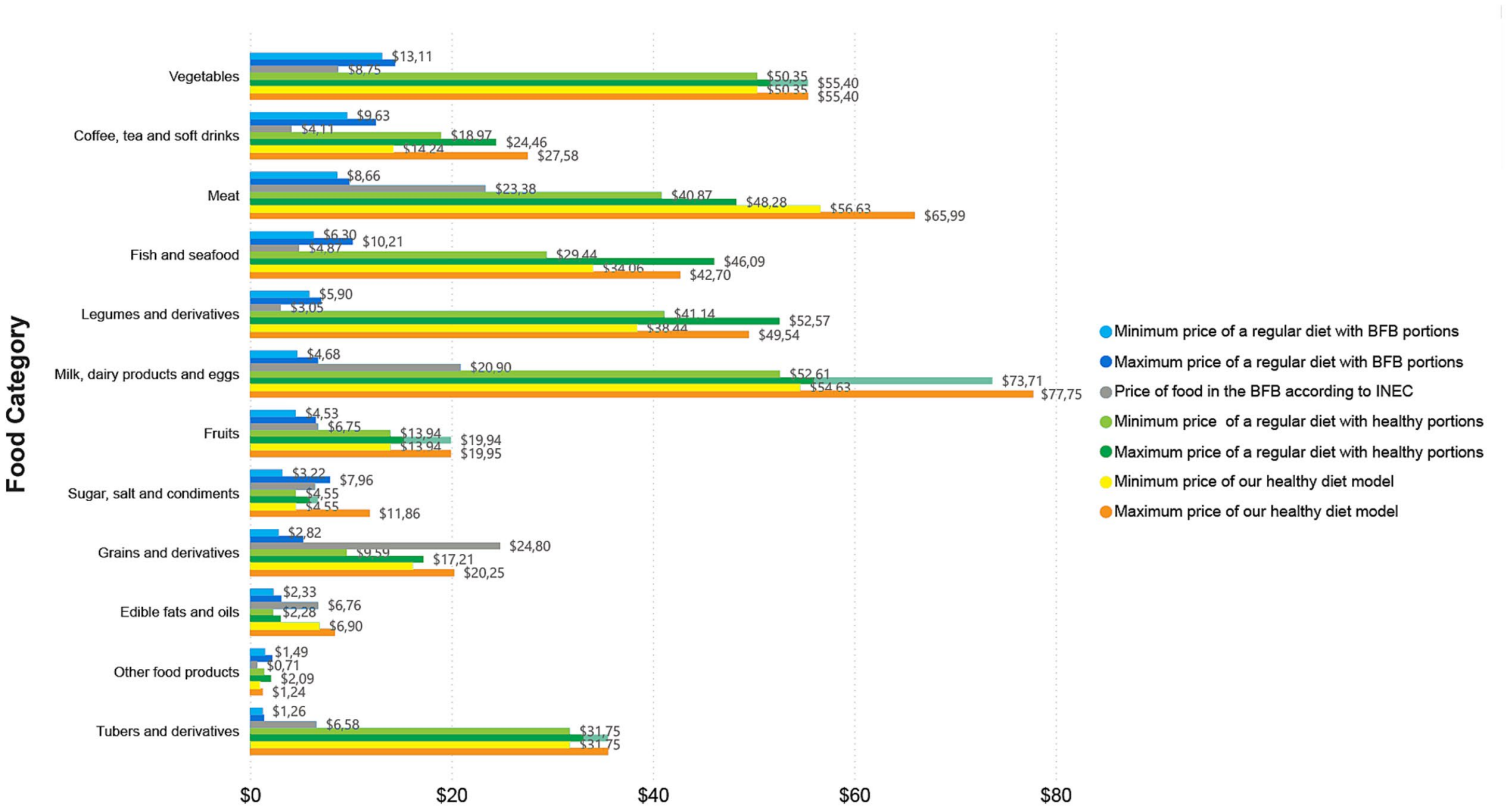
Price by food category in Guayaquil, showcasing minimum and maximum cost of the three analysed diets per month for two adults.

**Figure 4:**
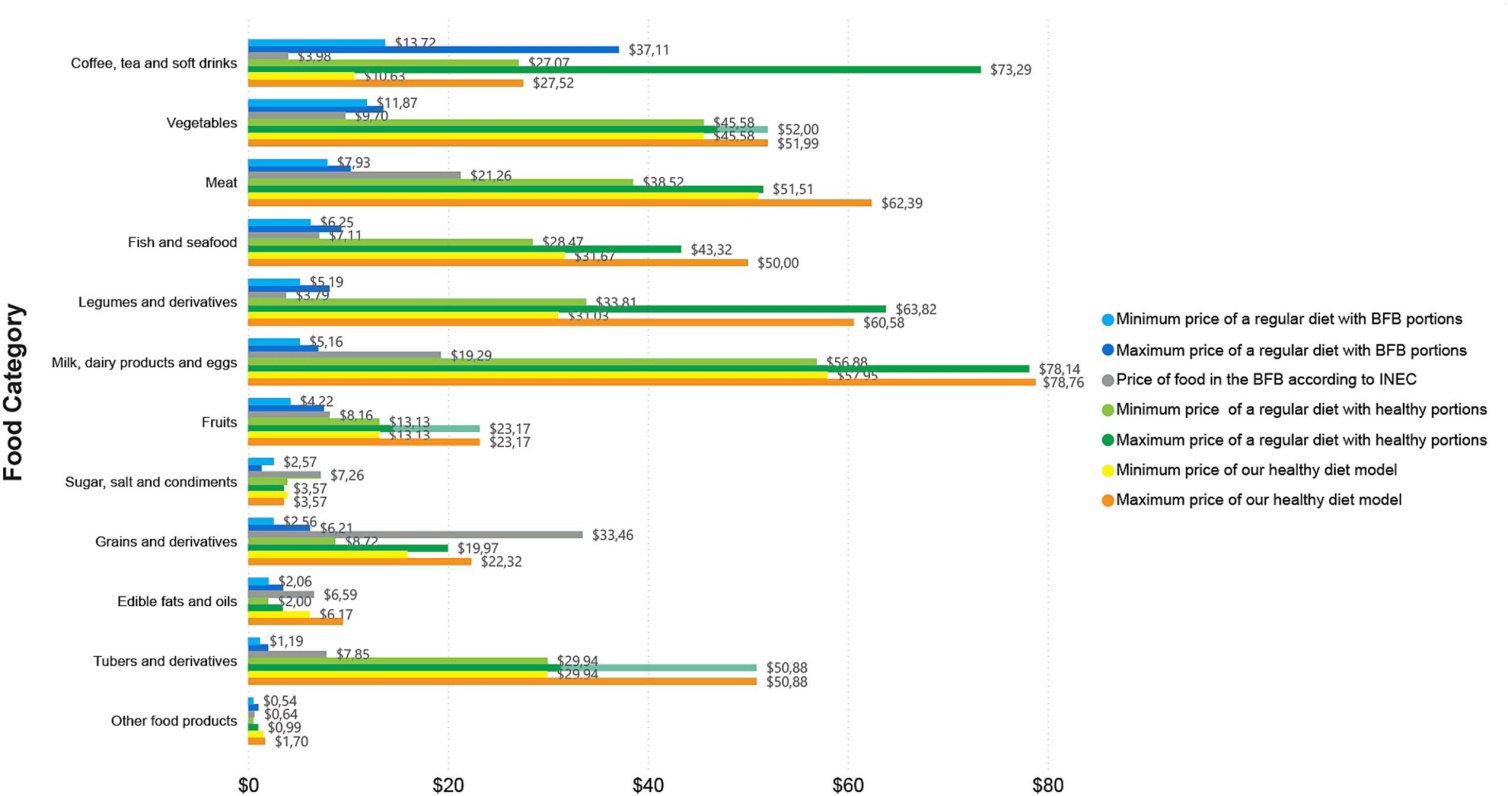
Price by food category in Quito, showcasing minimum and maximum cost of the three analysed diets per month for two adults.

In terms of the analysis by food category, Figures 2–4, report the prices overall, in Guayaquil, and in Quito, respectively. Each figure shows the price of a regular diet reported in the BFB by the INEC; the minimum and maximum cost of the regular diet with BFB portions; the minimum and maximum cost of the regular diet with healthy portions; and the minimum and maximum cost of our healthy diet model. All costs are based on diets for two adults for one month.

The most expensive category of the regular diet reported in the BFB by the INEC was “grains and derivatives” with an overall price of $28.29 ($24.80 for Guayaquil and $33.46 for Quito) followed by “meats” with $20.66 ($23.28 for Guayaquil and $21.26 for Quito) (Figures 2–4); “grains and derivatives” were more expensive in Quito (Figure 4), while “meats” were more expensive in Guayaquil (Figure 3). In contrast, when looking at our collected data from the supermarkets, we divided food prices into minimum and maximum cost; in the minimum cost (overall price) group of the regular diet with BFB portions, the most expensive food category was “vegetables” ($12.47) and “coffee, tea and non-alcoholic beverages” ($11.49) (Figure 2). The same categories were the most expensive when looking at the minimum and maximum cost of the regular diet with BFB portions both in Guayaquil and in Quito; in the latter, however, “coffee, tea and non-alcoholic beverages” were more expensive than “vegetables” (Figures 2,3).

When looking overall price at the regular diet with healthy portions at minimum cost, the most expensive categories were “milk, dairy products and eggs” with $54.75 followed by vegetables with $47.88 (Figure 2); the same occurred in Quito ($56.88 and $45.58, respectively), while in Guayaquil the most expensive was still “milk, dairy products and eggs” with $52.61, but followed by vegetables with $50.35 (Figures 2,3). The maximum cost for a regular diet with healthy portions kept the category “milk, dairy products and eggs” as the most expensive overall with $75.93, followed by “legumes and derivatives” with $58.20 (Figure 2); in Quito and in Guayaquil “milk, dairy products and eggs” were the most expensive with $78.14 and $73.71, respectively (Figures 2,3). In Guayaquil, “vegetables” took the second place with $55.40, while in Quito, “coffee, tea and non-alcoholic beverages” were the second most expensive with $73.29, (Figures 2,3).

On the other hand, when assessing our healthy diet model, a similar trend appeared, with “milk, dairy products and eggs” being the most expensive category (in both minimum and maximum cost) overall and for Quito (Figures 2,4); “meats” represented the second most expensive category (in both minimum and maximum cost) overall and in Quito, while being the most expensive (when looking at the minimum cost) and the second most expensive (when looking at maximum cost) in Guayaquil (Figure 3). Finally, in Guayaquil, the second most expensive category, in our minimum cost analysis was “milk, dairy products and eggs” with $54.63 and the first most expensive with $77.75 when looking at maximum cost (Figure 3).

In our comparison of diets, we noted that the sole category with a higher cost, as indicated in the BFB by the INEC, was “grains and derivatives.” Its prices exceeded those of this category in both the regular diet with healthy portions and our healthy diet model. This trend was evident in both the overall analysis and in individual cities (Quito and Guayaquil). In the remaining categories, the cost of a regular diet with healthy portions or our healthy diet model consistently exceeded the prices recorded in the BFB by the INEC. Furthermore, “legumes and derivatives” constituted the sole category where the expense of a regular diet in healthy portions exceeded that of our healthy diet model; conversely, in all other categories, the cost of our healthy diet model surpassed that of the regular diet with healthy portions (Figures 2–4).

Regarding overall food expenses, when looking into individual food items required per month for two adults, in the regular diet with BFB portions at minimum cost, the priciest items were coffee ($11.40) and fish ($5.65), while at maximum cost, they were $23.43 and $8.83, respectively. In the regular diet with healthy portions, the most expensive items at minimum cost were cheese ($24.76) and coffee ($22.56), while at maximum cost were coffee ($46.39) and cheese ($37.20). In our healthy diet model, the priciest items were chicken at $27.63 and boneless beef at $26.12 (when looking at the minimum cost), whereas the most expensive items in the maximum cost analysis were cheese at $37.20 and fish at $32.74.

**Figure 5:**
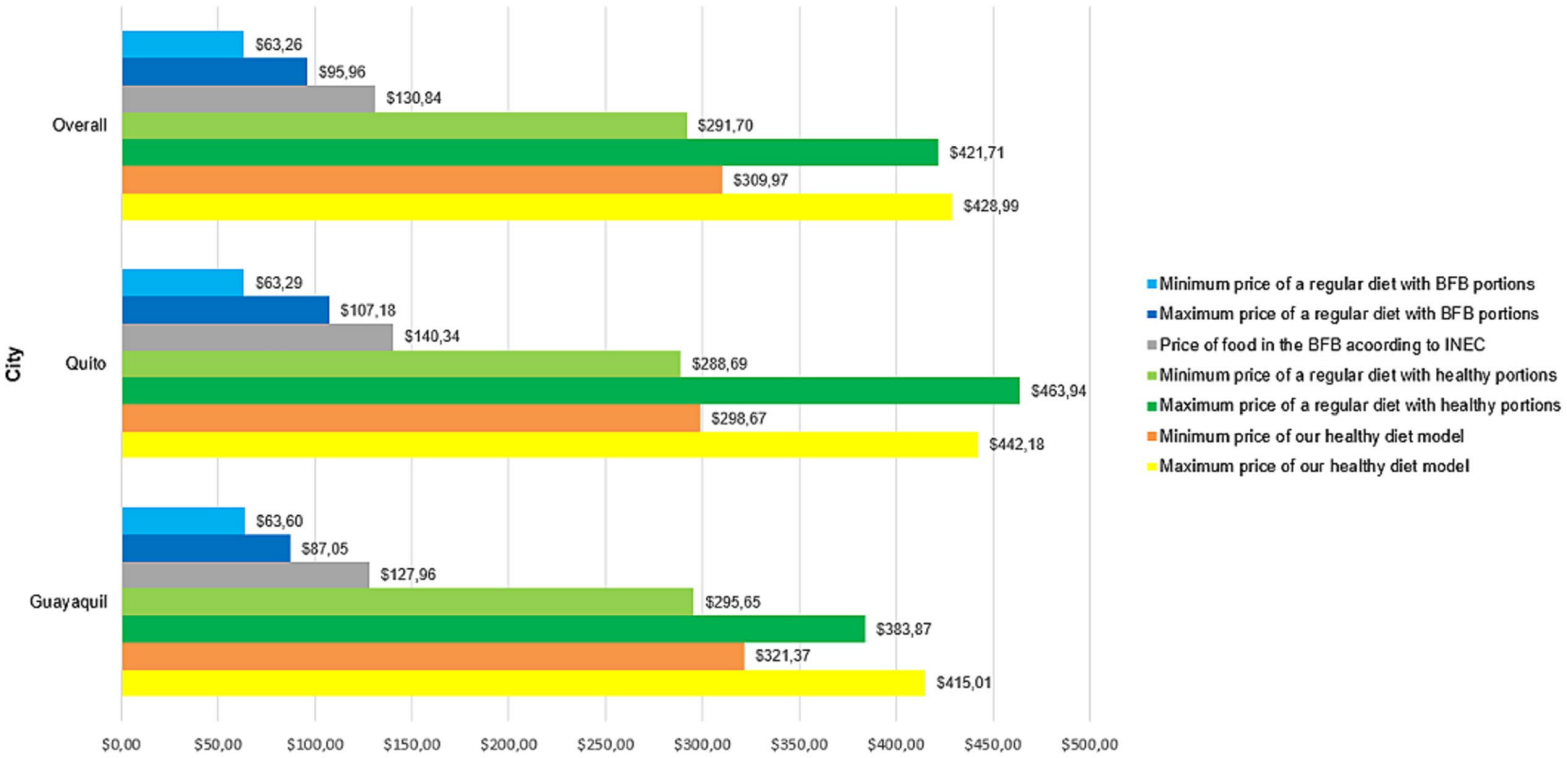
Total price, in general and by city, showcasing minimum and maximum cost of the three analysed diets for two adults.

In general, the cost of the regular diet per month for two adults with the portions recommended in the BFB was lower than the other healthier diets, with the minimum cost being $63.26 and the maximum cost being $95.96; the same pattern can be seen when looking at each city (Figure 5). However, when analysing the same regular diet per month for two adults but in healthy portions, a significant increase is seen, with the minimum and maximum costs raising to $291.70 and $421.71, respectively. In turn, when comparing this latter diet with our healthy diet model, there is not much difference in terms of the maximum cost (regular diet with healthy portions: $421.71; healthy diet model: $428.99); but there is a difference of $18.27 when comparing the minimum cost (regular diet with healthy portions: $291.70; healthy diet: $309.97) (Figure 5). Interestingly, in Quito, the maximum cost of our healthy diet model was lower than the maximum cost of the regular diet in healthy portions ($442.18 and $463.94, respectively) with a difference of $21.76 (Figure 5). In contrast, in Guayaquil, the difference between our healthy diet model and the regular diet with healthy portions is greater, with the minimum cost of the regular diet with healthy portions being $295.65 and that of our healthy diet model being $321.37, that is, a difference of $25.72; while at the maximum cost the difference was $31.14 (Figure 5).

It should be noted that diets were more expensive in Quito than in Guayaquil, except in minimum cost analysis of our healthy diet model, which was $298.67 in Quito and $321.37 in Guayaquil (Figure 5). A healthy diet in Guayaquil costs on average $368.19 for two adults per month ($184.10 per person), while in Quito the average is $370.43 for two adults per month ($185.22 per person). Our overall analysis revealed that the cost of the proposed healthy diet model was $184.74 per person per month. In this sense, a healthy diet per month for two adults in Guayaquil is $240.23 more expensive than the budgeted amount reported in the BFB ($127.96, adjusted for two people), while in Quito an extra $230.09 is required to eat healthy (per month for two adults) on top of the reported $140.34in the BFB adjusted for two people.

Additionally, the average daily cost of a healthy diet per person in Guayaquil was $6.14 and in Quito it was $6.17. While the daily cost of alimentation stipulated by the BFB is $2.13 in Guayaquil and $3.83 in Quito which represents $4.01 and $2.34 daily additional, respectively. Moreover, considering that the unified basic salary of Ecuador in 2023 was $450.00 (28), a person from Guayaquil or Quito would need to spend 41% of their wages to access a healthy diet; much more than the calculated 14.5% of the BFB. Even if we consider the average of the regular diet with healthy portions, an Ecuadorian worker would have to invest 39.63% of the unified basic salary to obtain it (Figure 5).

Finally, to incorporate the socioeconomic factor, we calculated the percentage of income required to afford a healthy diet according to income quintiles in Quito for the year 2022 (this information is not available for Ecuador as a whole or for 2023) (29). In this regard, a person in the first quintile (Q1) would need 378% of their income ($49) to afford a healthy diet; for the second quintile, that percentage would be 185.22% (based on an income of $100) and for the third quintile, 120.27% (income of $154) (29). Individuals in the fourth quintile (income: $244) would need to allocate 75.91% of their incomes, which also exceeds the range of affordability an affordable diet must represent less than 30% of the income (27). Consequently, only people in the fifth quintile (income: $658) can afford a healthy diet, spending 28.15% of their income (29).

## 4 Discussion

Our study determined that, in Ecuador, acquiring a healthy diet is more expensive than the reported regular diet in the Basic Family Basket (BFB) created by the Ecuadorian National Institute of Statistics and Census (INEC); it represents an investment of 41% of the unified basic salary of $450. The observed differences in cost of each diet are related to the nutritional quality ant the quantity of the food items required to meet nutritional requirements. In this regard, the healthy diet model includes higher-quality and less processed food, which generally have a higher price in our context. For instance, in the meat category, the healthy diet model includes skinless chicken and beef loin cuts, in line with international recommendations that promote lean protein intake and limit high-fat animal products. Another example is the used of semi-skimmed milk and cold-pressed olive oil in dark glass bottle in the healthy model to improve dietary quality; however, these items are more expensive than the refined and ultra-processed versions included in the BFB. Furthermore, the portion sizes in the healthy model are adjusted to align with the GABA guidelines and international recommendations, in order to provide a diet for two average adults with an energy requirement of 2000 kcal per day each one. This involves increasing the frequency or quantity of certain food categories. Although these changes offer nutritional benefits, they also contribute to increases cost of the healthy diet. On the other hand, our study reveals that only people in the highest income quintile can afford the healthy diet model. This highlights the economic challenges faced by average Ecuadorian households in accessing nutritionally adequate diets in a context of food insecurity and income inequality.

This is not the first report of the cost of healthy eating in the South American or Hispanic region. For instance, Verdugo et al. made a comparison between a healthy diet according to the Chilean food-based dietary guidelines and an unhealthy diet, using the minimum prices taken from a list established by the retail price regulatory agencies of their country in 2015; they determined that, a healthy diet was significantly more expensive than the unhealthy option (*p* < 0.001) even when corrected by caloric density (the unhealthy option had a lower cost per kilocalorie than the healthy option) (30). Similarly, Bouzas et al. (31) in their 6-year, parallel-group randomized clinical trial that included 6,838 Spanish adults with metabolic syndrome, found a directly proportional relationship between the quality of a diet (and its potential benefits) and its price; the higher the price, the greater the intake of healthy foods such as vegetables, whole grains and fruits, whereas the most economical diets were characterized by higher energy density foods (i.e., unhealthy food with higher kilocalories).

The cost of our healthy diet model in Ecuador for one person, as of September 2023, is $184.74 per month ($6.16 daily), this value is, approximately, two times higher than that published in June 2022 by the local newspaper “Primicias” that reported a value of $87.90 (32). This newspaper based its article on the report entitled “The State of Food Security and Nutrition in the World 2022” published by the Food and Agriculture Organization of the United Nations (FAO) (33); in this report, the calculated global daily cost of a healthy diet was $3.54, so that approximately 3.1 billion people, globally, cannot acquire an adequate diet due to this economic constrain (33). In addition, in Latin America and the Caribbean the cost was higher, at $3.89, being the region with the highest cost in acquiring a healthy diet (our calculated diet was $2.27 more expensive) (33). In comparison to this report, our study takes into account local variations in food prices across the two major cities in Ecuador, whereas the global report by FAO provides an average cost of healthy diets that may not accurately reflect the actual expenses faced by Ecuadorian households; furthermore, their report used purchasing power parity (PPP) dollars to compare the acquisition of goods between countries (33), which may not entirely represent the real expenditure in local currency (34, 35). The difference between this report and our study suggests that the cost of a healthy diet in Ecuador may have increased substantially in the past years, mirroring trends observed globally where the prices of nutritious foods have risen at a faster pace than those of less healthy options (36). This rapid increase in the price of healthy foods relative to less healthy alternatives exacerbates the already significant financial barriers faced by low-income populations in accessing a nutritious diet (37, 38). Regardless of the source, it is clear that accessing a nutritious, sustainable, and healthy diet represents a substantial economic burden for the average Ecuadorian employee (33, 35). It is essential to note that our study focused only on the prices of food items, without considering other associated costs such as preparation, storage, or transportation that could further increase the total expenditure required for a healthy diet.

Other studies have analysed the cost of healthy eating in a similar fashion as ours. For instance, Lee et al. conducted a study in Sydney and Canberra with data collected from November to December 2015; they divided the population into socioeconomic quintiles, with the first quintile being the families with the lowest income and fifth quintile being the families with the highest income. They reported that food was more expensive in Sydney compared to Canberra and that a regular diet was more expensive than a healthy one; also, families in the lowest quintile had greater difficulties in acquiring healthy food (27, 39). Another study done by Bracci et al. (40), in the same country, comparing the usual western diet, the diet based on dietary guidelines, and the Mediterranean diet between October and November 2022, determined that all the diets studied were affordable for the population considering that a typical person (single woman aged 30) earns AUD$1,835 per week and that the costs of the analysed diets ranged from AUD$75–80 (40). The aforementioned studies showed that healthy eating is affordable in Australia, which is not surprising given that the median weekly earnings are AUD$1300 (AUD$5200 per month), and the minimum wage, as of 2024, is AUD$915.9 weekly (AUD$3663.6 per month) 8 times higher than the basic monthly salary for an Ecuadorian employee (USD$450) (41, 42). In contrast, Van et al. conducted a study in several regions of Vietnam, based on the Vietnamese healthy dietary guidelines and extracting prices from national and regional databases from 2016 to 2020 (43). They concluded that, although acquiring this diet has been more feasible over the years included in the analysis, the acquisition gap of the population in the lowest socioeconomic quintile has remained unsustainably high (on average 68.4% of this group cannot acquire a healthy diet) (43). Finally, Rao et al. in their systemic review analysing healthier foods and diets from 27 articles written in English and published until 2011, the difference between healthier and less healthier options was $1.49, denoting that, although the gap is smaller than in our article, the healthy diet remains more expensive than the usual one (36). In Ecuador, as of April 2023, the rate of unemployment was 4.2%; however, only 35.2% of those employed earn the same as or higher than the unified basic salary (UBS) ($450), 50.2% earn less than the UBS, and 10.4% are employed but receive no salary according to the INEC (44). Meaning that, in Ecuador, 64.8% of the population could not afford a healthy diet, as of April 2023.

The analysis by supermarkets highlights the significant influence the retail environment has on the affordability of a diet. The healthy diet model was the most expensive across all supermarkets, reinforcing the economic challenges associated with adopting an adequate diet in Ecuador. The supermarket classified as low-end offered the lowest prices across all diet types (in minimum price), making it more affordable for low-income populations. Nevertheless, even in this store, the cost of a healthy diet was substantially higher than the regular diets. On the other hand, regular diet with BFB portions was cheaper in the mid-range and low-end supermarket. This may reflect limited nutritional quality but greater affordability. Moreover, the high-end supermarket has the highest minimum cost for the healthy diet model, raising concerns about the affordability of healthier options.

### 4.1 Limitations

One of the limitations of this study is that it is a cross-sectional analysis, so the prices were only collected at a single point in time, which may not reflect variations throughout the year or over time. Additionally, the prices were obtained from major supermarket chains, which may not represent the full range of food prices available in the cities studied. Another limitation is that the study did not take into account factors that may influence food prices, such as seasonality, transportation costs, or local market dynamics. For instance, it may be necessary to include local markets, community fairs, or bulk-buying options, where prices could be considerably lower. In this regard, the study may have overestimated the actual cost of a healthy diet. Moreover, this study proposed a healthy diet model based on the nutritional requirements for two healthy adults, without considering specific diseases or conditions, or the dietary needs associated with each life stages such as childhood, adolescence and older adulthood. Consequently, these findings may not be generalizable to all Ecuadorian households. Future research should incorporate specific dietary requirements of families with children or relatives with special nutritional needs to provide a realistic understanding of the affordability of a healthy diet in Ecuador.

Finally, the dietary adjustment does not account for the fibre content of the foods used, due to the absence of this information in the “Ecuadorian Food Exchange List” from the Ecuadorian dietary guideline. In addition, no adjustments were made for yield and food waste between the purchase and consumption, especially for fruits and tubers. Nevertheless, for the determination of the nutritional contribution, raw versions of protein sources and cooked versions of carbohydrates sources were used. Even though this affects the weight of the food, it does not significantly influence the nutritional value of the food items.

## 5 Conclusion

Our study underscores the substantial discrepancy in the affordability of regular vs. healthy eating in Ecuador, especially in its principal cities, Quito and Guayaquil. The results indicate that nutritious diets are consistently pricier than conventional diets. The regular diet with quantities recommended by the Basic Family Basket (BFB), which does not provide sufficient nutrients to be considered healthy, is significantly more economical than both a healthier variant of the standard diet and our suggested healthy diet model. The examination of food categories indicates that the most expensive components of a balanced diet are generally milk, dairy products, and eggs, succeeded by meats and vegetables, with notable price discrepancies between Quito and Guayaquil. The study also revealed that the financial strain of obtaining a healthy diet is significant, necessitating almost 41% of the unified basic salary (UBS), much above the 14.5% projected for a standard diet by the BFB; considering that 64.8% of the population earn less than the UBS, healthy eating in Quito and Guayaquil is not feasible.

This economic limitation is not exclusive to Ecuador; analogous findings from other locations suggest that the expense of nutritious diets is a worldwide concern, particularly for low-income demographics. The elevated cost of healthy foods intensifies the difficulties faced by many Ecuadorians, especially considering the country’s income inequalities. The study highlights the pressing necessity for governmental initiatives to enhance the accessibility and affordability of nutritious meals for all Ecuadorians, in light of these financial obstacles.

Furthermore, our findings highlight the pressing need for governmental action to reduce the affordability gap. First, subsidies or tax exemptions for essential healthy foods (such as dairy, lean proteins, and vegetables) could alleviate costs for consumers. Second, policies to strengthen local food systems and support direct trade between producers and consumers may reduce intermediaries and lower final prices. Third, integrating affordability targets into the existing Ecuadorian Food-Based Dietary Guidelines (GABA) would help align nutritional recommendations with socioeconomic realities. Finally, urban planning strategies, such as incentivising community markets and public procurement of local produce, could enhance accessibility of healthy foods for vulnerable groups. Finally, in terms of research, future studies should consider the environmental impact in the cost of different diets. Additionally, future investigations could explore the long-term health outcomes associated with both the regular and healthy models. It is also relevant to consider consumer behaviour and food preferences when adjusting the healthy diet, because these factors can influence its adoption. Integrating affordability and cultural acceptability into the healthy diet model is essential to address both undernutrition and the increasing prevalence of non-communicable diseases in low- and middle-income countries.

## Data Availability

The datasets for this article are not publicly available due to concerns regarding participant/patient anonymity. Requests to access the datasets should be directed to the corresponding author.
